# Role of Vitreous Detachment in Epiretinal Membrane Peeling: A Multimodal Imaging and Microperimetry Study

**DOI:** 10.3390/jcm13123565

**Published:** 2024-06-18

**Authors:** Federica Serino, Fabrizio Gaetano Saverio Franco, Daniela Bacherini, Marco Lupidi, Stefano Gallio, Claudio Esposito, Gianni Virgili, Cesare Mariotti, Fabrizio Giansanti

**Affiliations:** 1 Eye Clinic, Neuromuscular and Sense Organs Department, Careggi University Hospital, 50134 Florence, Italy; fabriziogsfranco@gmail.com (F.G.S.F.); daniela.bacherini@gmail.com (D.B.); gianni.virgili@unifi.it (G.V.); fabrizio.giansanti@unifi.it (F.G.); 2 Department of Neurosciences, Psychology, Drug Research and Child Health, University of Florence, 50121 Florence, Italy; stefano.gallio@unifi.it (S.G.); claudio.esposito@unifi.it (C.E.); 3 Eye Clinic, Department of Experimental and Clinical Medicine, Polytechnic University of Marche, 60121 Ancona, Italy; m.lupidi@staff.univpm.it (M.L.); mariottiocul@gmail.com (C.M.)

**Keywords:** epiretinal membrane, vitreous, posterior vitreous detachment, pars plana vitrectomy

## Abstract

**Background**: To investigate anatomical and functional changes of the macula caused by epiretinal membrane (ERM) peeling procedures in patients with or without posterior vitreous detachment (PVD). **Methods**: This is a multicentric prospective observational study on thirty-seven (37) patients affected by symptomatic ERM who underwent 25-gauge pars plana vitrectomy (PPV), induction of a PVD (as needed) and peeling of both the internal limiting membrane (ILM) and ERM. Optical coherence tomography–angiography (OCT-A) (*RS 3000*, Nidek, Japan) and microperimetry (*MP-3*, Nidek, Japan) were performed; central retinal thickness (CRT), foveal avascular zone (FAZ) area and perimeter, vessel density and perfusion density, retinal sensitivity and fixation stability (as a total mean retinal sensitivity (MRS), and MRS in the ellipse area and bivariate contour ellipse area (BCEA)) were recorded at baseline and up to postoperative month 3. **Results**: Eyes were classified as having complete PVD (51.4%) or incomplete PVD (48.6%). At baseline, patients with incomplete PVD had worse best-corrected distance visual acuity (BCDVA), total MRS, MRS in the ellipse area and BCEA, and higher CRT than patients with complete PVD. At month 3, the differences in BCDVA between the two groups remained statistically significant, with patients with incomplete PVD having worse results (difference: 0.199 logMAR, *p* < 0.001). The difference in the MRS in the ellipse area was statistically significant at month 3 (−3.378 Db, *p* = 0.035), with greater improvement in patients with complete PVD. **Conclusions**: Our study shows that patients with incomplete PVD have worse conditions at baseline than patients with complete PVD, and the differences in visual acuity and retinal sensitivity were maintained postoperatively.

## 1. Introduction

Idiopathic epiretinal membrane (ERM) is a relatively common pathological condition: the pooled age-standardized prevalence estimated is up 9.1% [1]. The condition is characterized by the constitution of a transparent, avascular, fibrocellular membrane on the inner retinal surface that adheres to and covers the internal limiting membrane (ILM) of the retina. Proliferation of glia, retinal pigment epithelium (RPE), or hyalocytes at the vitreoretinal interface after an anomalous posterior vitreous detachment is thought to cause ERM formation.

Surgical treatment of symptomatic patients consists of pars plana vitrectomy (PPV) and membrane peeling, eventually removing ILM. Sometimes, induction of a posterior vitreous detachment (PVD) is required before the membrane’s removal. In fact, PVD is present in around 80% of patients with idiopathic ERM [2]. The presence of an incomplete PVD may determine a more technically difficult procedure, with a complexity of forces which acts peripherally, increasing the risk for iatrogenic retinal breaks [3,4]. Less known is the effect of having an incomplete PVD, intraoperatively verified, on post operative outcomes. 

In certain cases of incomplete PVD, the vitreous body may be firmly attached to certain area of the macula region, mainly to the foveola and the margin of the fovea. Forces caused by either ERM or ILM peeling are known to induce functional and circulatory changes in the foveal region [5,6]. This study aims to describe the anatomical and functional changes of the macula after ILM peeling and PPV for ERM, using microperimetry and multi-modal imaging, in patients with complete or incomplete PVD. Particularly, we aimed to investigate focal loss of sensitivity and vasculature caused by peeling procedures in patients with or without PVD.

## 2. Materials and Methods

This is a multicentric prospective observational study on thirty-seven (37) patients affected by symptomatic ERM who underwent 25-gauge PPV, induction of a PVD (as needed) and peeling of both the ILM and ERM. The study was conducted at the Eye Clinic, Neuromuscular and Sense Organs Department, Careggi University Hospital (Florence, Italy), and at the Eye Clinic, Polytechnic University of Marche (Ancona, Italy). It was performed according to the current version of the Declaration of Helsinki (52nd WMA General Assembly, Edinburgh, Scotland, UK, October 2000). All the patients included in the study signed written informed consent to participate. The study was approved by the Careggi University Hospital Research Ethics Board. Exclusion criteria were other ocular pathology affecting central vision; glaucoma with significant perimetric involvement; history of intraocular surgery (except for cataract surgery); and lack of fixation or cooperation during the exam. We excluded cases with vitreomacular traction syndrome on OCT examination. Patients lost to follow-up were excluded from data analysis.

Baseline visit included a complete clinical assessment with best-corrected visual acuity (BCVA) recording, optical coherence tomography (OCT) and OCT–angiography (OCT-A) (*RS 3000 Advance 2*, Nidek, Japan); 3 × 3 OCT-A images were segmented automatically. Three of the authors (F.S., C.E. and S.G.) reviewed the accuracy of segmentations, and low-quality images (i.e., poor fixation) were excluded from the analysis. According to their morphologic features on the preoperative OCT, ERMs were classified in 4-stage scheme, as reported by Govetto [7]. Central retinal thickness (CRT), foveal avascular zone (FAZ) area and perimeter, vessel density (defined as the percentage of the total area occupied by vessels), and perfusion density (defined as the total area of perfused vasculature per unit area in a region of measurement) either of superficial (SVP) or deep vascular plexi (DVP) were recorded. CRT was measured manually with the caliper function of the Nidek OCT from the external limiting membrane (ELM) to the ILM, measured at the point of intersection of 6 radial lines on the scan [8]. FAZ area and perimeter vessel, and perfusion density were automatically calculated using the default RS-3000 Advance 2 Software (Version 1.12.0.13) [6]. Fixation stability is measured as the precision of eye fixation when one fixates intently on a stimulus for a certain period of time; it is a fundamental component of visual performance, and it is usually impaired in patients with macular disease. Retinal sensitivity and fixation stability were analyzed with microperimetry (*MP-3*, Nidek, Japan). Exams were performed under dim-light conditions with a dilated pupil in the study eye, while the fellow eye was patched. A grid of 33 spots was centered on the fovea region. The stimulus was a Goldmann III white spot with a stimulus duration of 200 ms. The threshold strategy was HFA 4-2, and background luminance was 31.4 asb. Stimulus attenuation ranged from 0 dB to 34 dB. Total mean retinal sensitivity (MRS) (10° circle), 95 bivariate contour ellipse area (BCEA) (as the area of an ellipse that encompasses 95.4% of fixation points), MRS in the ellipse area, and percentage of fixation points in a circle of 2° (p1) and 4° (p2) diameter were recorded.

Surgeries were performed by three of the authors (F.G., F.F. and C.M.). PVD status was assessed intraoperatively evaluating the configuration of vitreoretinal adhesions around the macula and optic nerve, using triamcinolone acetonide (TA) if needed. The observation of waves on retinal surface is considered a sign of attached posterior hyaloid [9]. When needed, PVD was inducted by engaging the attached posterior cortical vitreous with active suction of a 25-gauge cutter over the optic disc and elevating it towards the postero-anterior and peripheral direction away from the margin of the optic disc. Thus, patients were classified in two groups: complete PVD and incomplete PVD. No intraoperative complications were recorded. Post-operative therapy included antibiotic prophylaxis for 1 week (q.i.d.) and steroids (dexamethasone phosphate q.i.d.) for 2 weeks.

Patients were checked after the surgery on day 1, day 7, week 3 and month 3: these visits included a slit lamp examination of anterior and posterior segment of the eye (dilatated fundus exam) and assessment for post-operative complications. OCT, OCT-A and microperimetry were performed at month 1 and month 3.

Statistical analysis was performed using Stata 18.0 software (StataCorp, College Station, TX, USA). Linear, logistic, or ordinal (as appropriate) mixed models were used to compare the change in BCVA and retinal sensitivity between patients with or without PVD, with individuals as random effects to account for correlated data during follow-up and time as a random slope. BCVA data were converted to the logMAR scale. A *p* value of less than 0.05 was considered statistically significant.

## 3. Results

A total of 37 patients were included in our study (17 males, 20 females). All patients were symptomatic with metamorphopsia and/or blurred vision. The demographic and clinical characteristics of patients included in the study are summarized in Table 1. Eyes were classified as having complete PVD (19 eyes, 51.4%) or incomplete PVD (18 eyes, 48.6%), depending on intraoperative findings as defined in the Methods. Using ordinal logistic regression, we found incomplete PVD was more common in eyes with a more severe OCT stage (OR: 5.11, *p* = 0.021).

**Figure 1:**
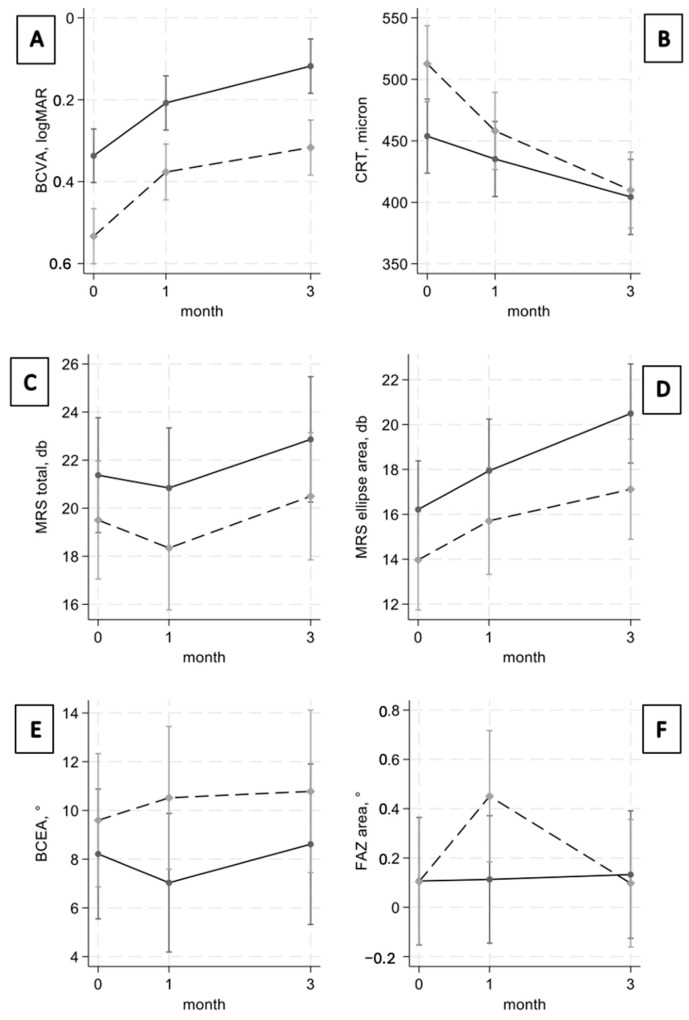
The six plots show the change in BCDVA (**A**), CRT (**B**), total MRS (**C**), MRS in ellipse area (**D**), BCEA (**E**), and FAZ area (**F**) during the follow-up for both the complete PVD group (solid line) and the incomplete PVD group (dashed line).

Figure 1 shows the change in BCDVA, CRT, total MRS and MRS in ellipse area, BCEA, and FAZ area during the follow-up for each PVD group. Table 2 presents the correspondent numerical data, together with the difference at each time point and the *p*-value of its significance. A progressive and significant improvement in BCDVA was recorded. The differences between the two groups were statistically significant and of a similar magnitude both at baseline and at post-operative months 1 and 3. Patients with incomplete PVD also showed greater mean preoperative CRT (513 µm) than patients with complete PVD (454 µm). The difference, which preoperatively was statistically significant (*p* = 0.008), was markedly reduced at the last follow-up. Both total MRS as assessed in the central 10 degrees of the retina and ellipse area showed an slight improvement overall, which was quite similar in the two groups. However, the difference of the MRS in the ellipse area became statistically significant at month 3 (*p* = 0.035), with greater improvement in patients with complete PVD. Patients with incomplete PVD had worse BCEA at baseline, which slightly worsened at month 3, whereas patients with complete PVD had stable results at the end of the follow-up period as compared to baseline. However, the differences between the two groups were never statistically significant. FAZ area remained stable in complete PVD groups, whereas it gradually enlarged in the incomplete PVD group up to month 1 before decreasing to values similar to the baseline. The differences between the two groups were never statistically significant.

**Figure 2:**
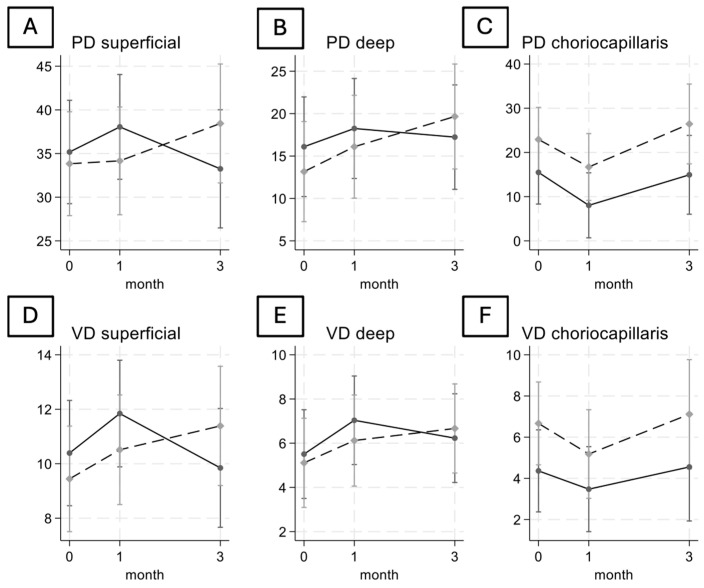
The six plots show the change in the perfusion density of the superficial vascular plexus (**A**), of the deep vascular plexus (**B**), and of the choriocapillaris (**C**); also shown is the vessel density of the superficial vascular plexus (**D**), of the deep vascular plexus (**E**), and of the choriocapillaris (**F**) during the follow-up for both the complete PVD group (solid line) and incomplete PVD group (dashed line).

Regarding the PD and VD of the SVP and DVP (Figure 2A, Figure 2B, Figure 2D and Figure 2E respectively), we observed that patients with complete PVD had an increase from baseline to month 1, but then both decreased. Instead, patients with incomplete PVD showed an overall increase in these values. The PD and VD of choriocapillaris (Figure 2C and Figure 2F respectively) remained stable at month 3 as compared to baseline. However, there were no statistically significant differences between the two groups.

## 4. Discussion

The exact role of PVD in the pathogenesis of ERM formation is still not clear. Historically, the presence of abnormal PVD was considered the etiopathogenetic factor for epiretinal fibrosis formation. However, more adherent hyaloid and incomplete PVD is occasionally found, meaning that ERM formation is a spectrum of disorders with variable surgical outcomes [10]. In these cases, induction of PVD is required, and the presence of a strong adherence between the vitreous cortex and retina may pose a significant intraoperative challenge.

The results of our study show that patients with incomplete PVD and more adherent posterior hyaloid have worse conditions at the baseline (lower BCDVA, higher CRT and overall, more severe OCT stage) than patients with complete PVD. We also found that visual improvement is similar after ERM removal, though the initial visual gap of about two Snellen lines between PVD groups remained unchanged at 1 and 3 months. On the other hand, CRT recovered to a similar extent in the two groups, with mean values around 400 microns at 3 months. Our findings confirm data published by Ota et al. [11], who previously retrospectively analyzed the relationship between PVD status and visual function in untreated patients affected by idiopathic ERM. They reported that patients with partial PVD with persistent vitreous attachment had worse BCDVA after 2 years. They theorized that chronic vitreous traction on the macula region may cause ILM cracking and glia cell migration, suggesting that early surgery is desirable in these cases. In our study, a worse visual outcome with PPV was consistent with the worse VA before surgery, despite successful ERM peeling and foveal anatomy reconstitution upon OCT examinations. In fact, previous studies [12,13] have shown that there is a linear correlation between preoperative OCT stages [7] and post-operative BCDVA, with later stages having a worse visual outcome as compared to early stages, even with statistically significant improvement in CRT in all stages. These studies did not consider PVD status in analyses.

We investigated further functional and anatomical aspects using microperimetry and multi-modal imaging. We found that at baseline, patients with incomplete PVD had lower MRS, both in the 10 central degrees and in the ellipse area, than patients with complete PVD. We also observed a slight decrease in MRS (both total and in the ellipse area) in the two groups at month 1; this may be due to the toxic effect of the dyes used during the peeling as well as the potential mechanical damage to the retina (even microscopic damage) because ILM is the basal lamina of the Müller cells. Nonetheless, there was an improvement in the two groups at the end of the follow-up. Microperimetry proved to be a useful diagnostic tool in evaluating ERM peeling outcomes [14]. Del Vecchio et al. demonstrated a significant improvement in retina sensitivity from baseline to 4 years after surgery for ERM. However, they did not categorize for PVD status. In our study, the differences that we observed in the two groups cannot simply be explained by the mechanical damage from forceps during the peeling (the more adherent the hyaloid is, the more pinching is needed) because these maneuvers are avoided in the very central fovea and performed in the perifovea. An alternative explanation is a potential effect of the worse OCT stages on consequent cell disorganization. This is consistent with a previous study which correlated OCT classification with MRS parameters of both the foveal and perifoveal area [15]. However, the role of the PVD induction is also to be considered. The vitreous cortex is attached superficially to the ILM, most firmly at the vitreous base, the optic disc margin, along major retinal vessels, and at the foveola in a 500 µm diameter plaque configuration which sometimes may persist after spontaneous PVD, suggesting firm attachment [16]. Analysis of retinochoroidal vascular changes did not show differences between the two groups. Both vessel and perfusion density of either superficial or deep plexi increased after surgery in the incomplete PVD group, as reported in a previous study [6]. The complete PVD group showed a blood flow improvement up to month 1, followed by a slight reduction to the baseline values at month 3. FAZ area was also similar in the two groups at baseline and the end of follow-up. Tangential forces associated with ERM resulted in vascular shift, but PVD induction did not seem to affect vascularity.

To best of our knowledge, this is the first longitudinal study investigating the effect of PVD status on surgery for ERM. The strength of this study is its prospective design conducted at two different clinics. Moreover, the results of analyses were consistent between centers. Its limitations are the small sample size and the relatively short follow-up period.

## 5. Conclusions

Our study found that incomplete PVD is associated with lower preoperative visual acuity compared to complete PVD, a gap that is maintained after surgery despite similar anatomic recovery. The pre-existence of posterior vitreous separation could be important in choosing surgical approach and timing. We suggest that surgeons consider removal of ERMs in the early stages in cases of incomplete PVD found upon preoperative evaluation.

## Figures and Tables

**Table 1:** Demographic and clinical characteristics of patients included in the study.

	Complete PVD (n.19)	Incomplete PVD (n.18)	Total (n.37)	*p*-Value
**Age (years),** mean value ± SD	73.1 ± 1.8	74.2 ± 1.7	73.6 ± 7.4	0.67 ^a^
**Combined cataract surgery**	13 (54.2%)	11 (45.8%)	24 (64.9%)	0.64 ^b^
**BCDVA (**logMAR**)**	0.4 ± 0.03	0.5 ± 0.5	0.4 ± 0.2	<0.001 ^a,^*
**OCT Stage (**Govetto classification**)**				0.021 ^c^
***2***	9 (47.4%)	3 (16.7%)	12 (32.4%)	
***3***	9 (47.4%)	10 (55.6%)	19 (51.4%)	
***4***	1 (5.2%)	5 (27.7%)	6 (16.2%)	
**CRT** (µm), mean value ± SD	453.8 ± 16	512.7 ± 21.0	482.5 ± 13.8	0.03 ^a^
**Total MRS** (dB), mean value ± SD	21.4 ± 1.5	19.5 ± 1.3	20.5 ± 1.0	0.35 ^a^
**MRS in Ellipse Area** (dB), mean value ± SD	16.2 ± 1.4	14.0 ± 1.4	15.1 ± 1.0	0.25 ^a^
**BCEA** ± SD	8.2 ± 1.7	9.6 ± 1.3	8.9 ± 1.1	0.52 ^a^
**FAZ area** ± SD	0.1 ± 0.02	0.1 ± 0.01	0.1 ± 0.01	0.93 ^a^

Abbreviations: SD: standard deviation; BCDVA: best-corrected distance visual acuity; OCT: optical coherence tomography; CRT: central retinal thickness; MRS: mean retinal sensitivity; BCEA: bivariate contour ellipse area; FAZ: foveal avascular zone. Symbols: ^a^ data obtained using *t*-test; ^b^ data obtained using chi square; ^c^ data obtained using ordinal logistic regression. * Threshold for Bonferroni-adjusted statistical significance: *p* < 0.005 (11 comparisons).

**Table 2:** BCDVA, CRT, Total MRS and MRS in ellipse area, BCEA, and FAZ area during the follow-up for each PVD group.

	Timing	Complete PVD	Incomplete PVD	Difference	*p*-Value
**BCDVA** (logMAR), (SE)	baseline	0.337 (0.033)	0.533 (0.034)	0.196 (0.048)	<0.001 *
	1 month	0.208 (0.034)	0.376 (0.035)	0.169 (0.049)	0.001 *
	3 months	0.118 (0.034)	0.317 (0.034)	0.199 (0.048)	<0.001 *
**CRT** (µm), mean value (SE)	baseline	453.8 (15.4)	512.7 (15.8)	58.825 (22.012)	0.008
	1 month	435.2 (15.6)	458.0 (16.0)	22.821 (22.360)	0.307
	3 months	404.4 (15.6)	409.9 (15.8)	5.569 (22.177)	0.802
**Total MRS** (dB), mean value (SE)	baseline	21.374 (1.219)	19.506 (1.252)	−1.868 (1.747)	0.285
	1 month	20.840 (1.277)	18.343 (1.314)	−2.496 (1.832)	0.173
	3 months	22.860 (1.330)	20.494 (1.350)	−2.365 (1.895)	0.212
**MRS in Ellipse Area** (dB), mean value (SE)	baseline	16.216 (1.105)	13.967 (1.136)	−2.249 (1.585)	0.156
	1 month	17.940 (1.177)	15.707 (1.213)	−2.233 (1.690)	0.186
	3 months	20.495 (1.136)	17.117 (1.136)	−3.378 (1.6)	0.035
**BCEA** (SE)	baseline	8.216 (1.359)	9.6 (1.387)	1.384 (1.948	0.477
	1 month	7.036 (1.451)	10.521 (1.495)	3.485 (2.083)	0.094
	3 months	8.615 (1.681)	10.783 (1.703)	2.169 (2.392)	0.365
**FAZ area** (SE)	baseline	0.107 (1.132)	0.104 (0.132)	−0.002 (0.187)	0.990
	1 month	0.113 (0.132)	0.451 (0.136)	0.337 (0.189)	0.075
	3 months	0.133 (0.132)	0.1 (0.132)	−0.035 (0.187)	0.851

Abbreviations: SE: standard error; BCDVA: best-corrected distance visual acuity; CRT: central retinal thickness; MRS: mean retinal sensitivity; BCEA: bivariate contour ellipse area; FAZ: foveal avascular zone. * Threshold for Bonferroni-adjusted statistical significance: *p* < 0.003 (18 comparisons).

## Data Availability

The data presented in this study are available on request from the corresponding author.

## References

[1] Xiao W., Chen X., Yan W., Zhu Z., He M. (2017). Prevalence and risk factors of epiretinal membranes: A systematic review and meta-analysis of population-based studies. BMJ Open.

[2] Chung S.E., Lee J.-H., Kang S.W., Kim Y.T., Lee S.W. (2011). Characteristics of epiretinal membranes according to the presence or absence of posterior vitreous detachment. Eye.

[3] Chung S.E., Kim K.-H., Kang S.W. (2009). Retinal breaks associated with the induction of posterior vitreous detachment. Am. J. Ophthalmol..

[4] Mura M., Barca F., Dell’Omo R., Nasini F., Peiretti E. (2016). Iatrogenic retinal breaks in ultrahigh-speed 25-gauge vitrectomy: A prospective study of elective cases. Br. J. Ophthalmol..

[5] Feng J., Yang X., Xu M., Wang Y., Shi X., Zhang Y., Huang P. (2021). Association of Microvasculature and Macular Sensitivity in Idiopathic Macular Epiretinal Membrane: Using OCT Angiography and Microperimetry. Front. Med..

[6] Bacherini D., Dragotto F., Caporossi T., Lenzetti C., Finocchio L., Savastano A., Savastano M.C., Barca F., Dragotto M., Vannozzi L. (2021). The Role of OCT Angiography in the Assessment of Epiretinal Macular Membrane. J. Ophthalmol..

[7] Govetto A., Lalane R.A., Sarraf D., Figueroa M.S., Hubschman J.P. (2017). Insights Into Epiretinal Membranes: Presence of Ectopic Inner Foveal Layers and a New Optical Coherence Tomography Staging Scheme. Am. J. Ophthalmol..

[8] Taban M., Sharma S., Williams D.R., Waheed N., Kaiser P.K. (2009). Comparing retinal thickness measurements using automated fast macular thickness map versus six-radial line scans with manual measurements. Ophthalmology.

[9] Martinez-Toldos Jose J., Hoyos Jairo E., Corcostegui B. (2013). Step by Step Vitrectomy.

[10] Bu S.-C., Kuijer R., Li X.-R., Hooymans J.M.M., Los L.I. (2014). Idiopathic epiretinal membrane. Retina.

[11] Ota A., Kakehashi A., Tanaka Y., Toyoda F., Shimmura M., Kinoshita N., Takano H. (2015). Relationship between variations in posterior vitreous detachment and visual prognosis in idiopathic epiretinal membranes. Clin. Ophthalmol..

[12] Momin S.N.A., Siddiqui M.A.R., Hashmi S., Memon A.S., Tayyab H., Choudhary R.A. (2022). Post-operative visual outcomes based on morphological staging of idiopathic epiretinal membranes on OCT. Int. J. Ophthalmol..

[13] González-Saldivar G., Berger A., Wong D., Juncal V., Chow D.R. (2020). Ectopic Inner Foveal Layer Classification Scheme Predicts Visual Outcomes after Epiretinal Membrane Surgery. Retina.

[14] Del Vecchio M., Lavia C., Nassisi M., Grignolo F.M., Fea A.M. (2016). Microperimetric Assessment after Epiretinal Membrane Surgery: 4-Year Follow-Up. J. Ophthalmol..

[15] Xu Z., Mao J., Lao J., Deng X., Liu C., Xu J., Wu S., Chen Y., Shen L. (2021). Macular Retinal Sensitivity and Microvasculature Changes before and after Vitrectomy in Idiopathic Macular Epiretinal Membrane with Classification. Ophthalmologica.

[16] Kishi S., Demaria C., Shimizu K. (1986). Vitreous cortex remnants at the fovea after spontaneous vitreous detachment. Int. Ophthalmol..

